# Real-world experience of teriflunomide in relapsing multiple sclerosis: paramagnetic rim lesions may play a role

**DOI:** 10.3389/fimmu.2024.1343531

**Published:** 2024-03-13

**Authors:** Hongmei Tan, Xiang Li, Yuxin Li, Fanru He, Jingzi ZhangBao, Lei Zhou, Liqin Yang, Chongbo Zhao, Chuanzhen Lu, Qiang Dong, Haiqing Li, Chao Quan

**Affiliations:** ^1^ Department of Neurology, Huashan Hospital, Shanghai Medical College, Fudan University, Shanghai, China; ^2^ National Center for Neurological Disorders, Shanghai, China; ^3^ Department of Radiology, Huashan Hospital, Shanghai Medical College, Fudan University, Shanghai, China; ^4^ Institute of Functional and Molecular Medical Imaging, Fudan University, Shanghai, China

**Keywords:** multiple sclerosis, magnetic resonance imaging, teriflunomide, paramagnetic rim lesion, no evidence of disease activity

## Abstract

**Objectives:**

The aims of this study were to report the effectiveness and safety of teriflunomide in Chinese patients with relapsing–remitting multiple sclerosis (RRMS) and to explore the association of paramagnetic rim lesion (PRL) burden with patient outcome in the context of teriflunomide treatment and the impact of teriflunomide on PRL burden.

**Methods:**

This is a prospective observational study. A total of 100 RRMS patients treated with teriflunomide ≥3 months were included in analyzing drug persistence and safety. Among them, 96 patients treated ≥6 months were included in assessing drug effectiveness in aspects of no evidence of disease activity (NEDA) 3. The number and total volume of PRL were calculated in 76 patients with baseline susceptibility-weighted imaging (SWI), and their association with NEDA3 failure during teriflunomide treatment was investigated.

**Results:**

Over a treatment period of 19.7 (3.1–51.7) months, teriflunomide reduced annualized relapse rate (ARR) from 1.1 ± 0.8 to 0.3 ± 0.5, and Expanded Disability Status Scale (EDSS) scores remained stable. At month 24, the NEDA3% and drug persistence rate were 43.8% and 65.1%, respectively. In patients with a baseline SWI, 81.6% had at least 1 PRL, and 42.1% had ≥4 PRLs. The total volume of PRL per patient was 0.3 (0.0–11.5) mL, accounting for 2.3% (0.0%–49.0%) of the total T2 lesion volume. Baseline PRL number ≥ 4 (OR = 4.24, *p* = 0.009), younger onset age (OR = 0.94, *p* = 0.039), and frequent relapses in initial 2 years of disease (OR = 13.40, *p* = 0.026) were associated with NEDA3 failure. The PRL number and volume were not reduced (*p* = 0.343 and 0.051) after teriflunomide treatment for more than 24 months. No new safety concerns were identified in this study.

**Conclusion:**

Teriflunomide is effective in reducing ARR in Chinese patients with RRMS. Patients with less PRL burden, less frequent relapses, and relatively older age are likely to benefit more from teriflunomide, indicating that PRL might be a valuable measurement to inform clinical treatment decision.

## Introduction

Multiple sclerosis (MS) is a chronic inflammatory and neurodegenerative disease of the central nervous system (CNS). Approximately 85% of the patients with MS begin with episodes of reversible neurological deficits, followed by progressive neurological deterioration over time (1). Preventing relapses and slowing disability progression are the main treatment goals of MS. However, the MS disease trajectory exhibits large variation (2). Along with the rapid expanding array of MS disease-modifying therapies (DMTs), a personalized approach to medication should be emphasized (3).

Teriflunomide (Aubagio^®^, Sanofi Genzyme) has been approved in China since 2018. Through selective and reversible inhibition of dihydroorotate dehydrogenase, it blocks *de novo* pyrimidine synthesis and reduces the proliferation of autoreactive lymphocytes (4). The efficacy and safety of teriflunomide in relapsing MS have been demonstrated in two pivotal phase 3 randomized control trials (RCTs) (5, 6) and long-term extension studies (7, 8). In previous Chinese real-life studies, teriflunomide was shown to reduce the annualized relapse rate (ARR) significantly, and the 12-month no evidence of disease activity (NEDA) 3 proportion was approximately 79% (9–11). However, Chinese data based on longer follow-up are lacking, and the association between novel magnetic resonance imaging (MRI) measurements and treatment outcome were rarely investigated.

Paramagnetic rim lesions (PRLs), an emerging imaging biomarker for MS, has iron-laden microglia/macrophages and reactive astrocytes at lesion edges inducing a rim of decreased signal on susceptibility-weighed imaging (SWI), reflecting chronic active inflammation in histopathology (12–16). PRL has the capacity to enlarge continuously over years (15, 17). Higher PRL numbers have been shown to strongly correlate with sustained tissue damage and greater clinical progression, especially progression independent of relapse activity (PIRA) (18–20).

In this study, we reported the real-life effectiveness of teriflunomide. More importantly, we explored the association between baseline clinical/MRI factors (including PRL) and patient outcome during teriflunomide treatment, and investigated the impact of teriflunomide on PRL burden.

## Methods

### Study population

This is a prospective, single-center, and observational study. From October 2018 to January 2023, 189 consecutive patients with MS initiating treatment with teriflunomide (Aubagio^®^, Sanofi Genzyme) were registered in the Department of Neurology, Huashan Hospital, the National Centre for Neurological Disorders (NCND) of China. Among them, 100 patients were included in this study, forming a “total cohort”. The inclusion criteria were (i) diagnosis of RRMS according to the 2017 McDonald criteria (21), (ii) treatment with teriflunomide ≥ 3 months, (iii) with clear and traceable disease history at inclusion, and (iv) compliance to the clinical disability and safety monitoring plan. The total cohort was used to present drug persistence and safety profile.

**Figure 1:**
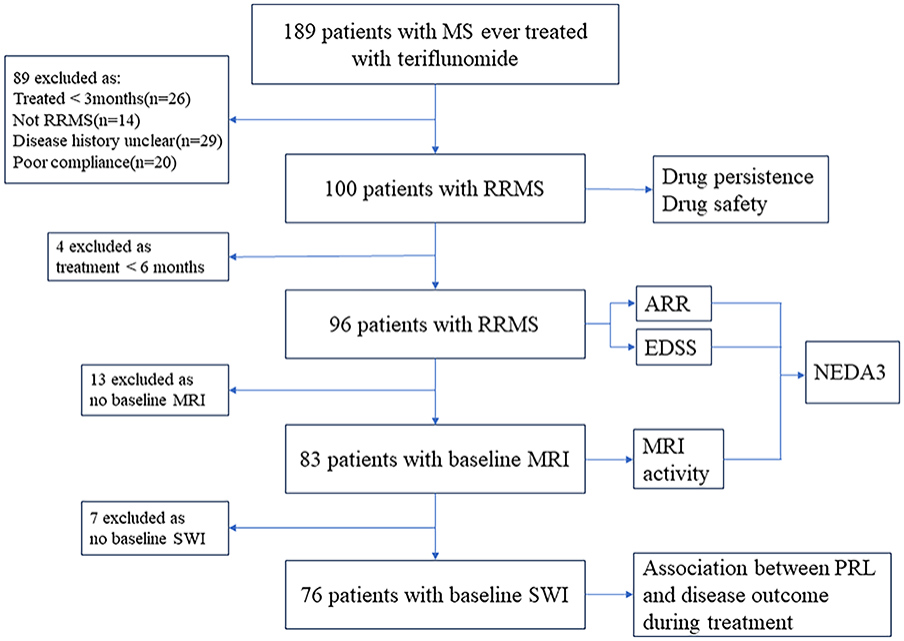
Flowchart of data processing and exclusions. MS, multiple sclerosis; RRMS, relapsing–remitting multiple sclerosis; ARR, annualized relapse rate; EDSS, Expanded Disability Status Scale; MRI, magnetic resonance imaging; NEDA, no evidence of disease activity; SWI, susceptibility-weighted imaging; PRL, paramagnetic rim lesion.

An “effectiveness cohort” was additionally defined from the total cohort that consisted of 96 patients on teriflunomide treatment over 6 months, for analysis of ARR and EDSS worsening, and some for NEDA3% (**Figure 1**).

Furthermore, data from 76 patients with baseline SWI were used to assess the association between baseline PRL burden and disease outcome during teriflunomide treatment.

Patients’ previous disease histories were collected from medical file review. Visits were scheduled at teriflunomide initiation (same as “at inclusion” or “baseline”), month 1, month 2, month 3, month 6, and then every 6 months after teriflunomide initiation for various purposes. Blood tests (such as blood cell counts and liver function) were performed at baseline, every month for the initial 3 months and then every 6 months. Expanded Disability Status Scale (EDSS) scores were assessed at baseline and every 6 months. Brain MRI was performed at baseline, month 6, month 12, and then every 12 months. In case any suspected relapse or worsening, additional visits (usually at the acute phase and at 90 days from the onset of a relapse) were arranged. The follow-up information was recorded prospectively and formed a data set, which was locked in June 2023. Statistical analyses were performed during June–August 2023.

This study was approved by the medical ethics committees of Huashan Hospital (HIRB-2020824). Written informed consent was obtained from each participant.

### Definition and outcome measures

The primary outcomes were ARR, EDSS score, MRI activity, and NEDA3. The secondary outcomes were drug persistence, adverse events (AEs), and the association of baseline PRL burden with patient outcome. The exploratory outcome was the change of PRL during treatment.

A relapse/attack was defined as any new MS-related neurologic symptom not associated with fever or infection lasting for at least 24 h and accompanied by new neurologic signs (22). Recovery from the onset attack was classified as complete if neurological signs or symptoms that developed during the onset attack became absent before the second attack (or EDSS score declined to ≤1.0); otherwise, incomplete recovery was considered.

Disability was characterized using EDSS scores. Confirmed disability worsening (CDW) was based on EDSS and defined by an increase in EDSS (≥1.0 point for patients with a baseline EDSS of 0–5.0 and by 0.5 points for patients with a baseline EDSS of ≥5.5) confirmed by an EDSS assessment 6 months apart from the onset of the worsening, and sustained till the last available visits (23). RAW was a CDW event with an EDSS worsening onset within 90 days from the onset of a relapse. PIRA was defined as a CDW event with either no prior relapse or an EDSS worsening onset more than 90 days after the start date of the last relapse, and no relapse occurs within 30 days before or after the EDSS worsening confirmation at 6 months (23, 24).

NEDA3 was fulfilled when clinical relapses, CDW sustained for 6 months, and new or enlarging T2 or T1 gadolinium (Gd)-enhancing lesions on MRI were absent (25).

### Brain MRI

MRI examinations were performed using a 3T scanner (Discovery MR750W, GE Medical System) with an 8-channel phased-array head coil. The parameters were as follows: (1) transverse FLAIR, TR = 8,000 ms, TE = 112.38 ms, TI = 2,352.85 ms, flip angle = 160°, and voxel size = 0.46×0.46×2.0 mm^3^; (2) transverse SWI, TR = 39.4 ms, TE = 22.576 ms, flip angle = 15°, and voxel size = 0.46×0.46×2 mm^3^; and (3) T1WI, transverse T1 BRAVO, TR = 8.528 ms, TE = 3.228 ms, TI = 400 ms, and voxel size = 1.0×1.0×1.0 mm^3^. Contrast-enhanced T1W1 scans were performed 10 min after intravenous Gd injection (flow rate, 1–2 mL/s; contrast dose, 0.1 mmol/kg). The phase images and magnitude images were automatically reconstructed.

We used the Statistical Parametric Mapping analysis package (SPM12, http://www.fil.ion.ucl.ac.uk/spm/software/spm12/) together with the Computational Anatomy Toolbox for SPM (CAT12, http://www.neuro.uni-jena.de/cat/) for voxel-based morphometry (VBM) analyses. T1-weighted images were bias-corrected, registered to the Montreal Neurological Institute (MNI) standard space, and segmented into gray matter, white matter, and cerebrospinal fluid by a multilevel procedure, and the total brain volume was finally calculated.

The Lesion Segmentation Tool (LST) (26) version 3.0.0 from the SPM software package was used to quantify the lesions based on FLAIR images. The output of the LST segmentation was then manually inspected and corrected, if needed, by experienced raters (YL and HL) using the Medical Imaging Interaction Toolkit (MITK) version 2023.04.2. MRICron version 1.0.20190902 was employed to calculate the total T2 lesion volume for each patient.

Two independent raters (LY and FH) used MITK to delineate PRLs on SWI-Phase images. NiftyReg was employed to rigidly align the FLAIR and SWI-Phase images, and the lesion masks were transformed to the SWI-Phase space using nearest-neighbor interpolation (27), with each lesion within the mask assigned a unique identification number. Criteria for a PRL were defined as a complete dark signal rim surrounding the lesion’s edge, showing hypointense compared to the lesion core and adjacent white matter, and the rim was visible on at least two consecutive slices. Before the start of the study, the raters (LY and FH) marked 20 scans and discussed any discrepancies. They then independently reviewed all the remaining cases (Cohen’s Kappa = 0.89), and in the case of any disagreements, it was adjudicated by two experienced radiologists (YL and HL). MRICron was used to calculate the total number and volume of PRL for each participant.

All the raters and radiologists were blind to the patients’ clinical situations.

### Statistical analysis

Statistical analyses were performed using SPSS, version 26.0 (SPSS Inc., Chicago, IL, USA) and R software, version 4.3.1 (R Foundation for Statistical Computing, Vienna, Austria). For descriptive analysis, continuous data were displayed as median with range/interquartile range (IQR) or mean ± standard deviation (SD), and categorical data were presented with counts and percentages. The Wilcoxon signed-rank test was used to assess the changes in the ARR and EDSS scores after teriflunomide treatment.

The times to the first relapse, CDW, MRI activity, and NEDA3 failure were investigated with the Kaplan–Meier method. Kaplan–Meier method was also applied to estimate the relative contributions of composite PIRA and RAW to CDW.

We investigated the association between patient outcome and the following factors: age at onset, sex, disease duration before treatment, relapses before treatment, relapses during the first 2 years of disease, regions involved at onset, recovery from first attack, baseline EDSS score, baseline PRL number and volume, baseline T2 lesion volume, and total brain volume. We set the cutoff of 4 for PRL number according to previous studies (18, 19). PRL volume, total T2 lesion volume, and total brain volume were dichotomized by cutoff values determined by the “surv_cutpoint” of the “survminer” R package. Multicollinearity between these factors were examined and excluded by multicollinearity diagnostic tests of SPSS. Univariate and multivariate logistic regressions were then performed. Factors with *p* < 0.2 in the odds ratio (OR) defined by univariate analysis were adopted into multivariate analysis. The false discovery rate was used in the correction of multiple testing (Benjamini–Hochberg procedure). ORs were reported with 95% confidence intervals (CIs). Statistical significance was set at a *p*-value of <0.05 (two-tailed).

## Results

### Demographics

From 2018 to 2023, 100 eligible patients with RRMS treated with teriflunomide over 3 months were included as the total cohort (**Figure 1**). Most of them (87%, 87/100) were treatment naïve. The baseline demographic and clinical data are shown in **Table 1**. The median age of MS onset was 26.9 (9.4–56.8) years, and 64% were female. The mean EDSS score at inclusion was 1.3 ± 1.4. The median duration on teriflunomide treatment was 19.7 (3.1–51.7) months.

**Table 1:** Baseline data of the 100 patients with RRMS treated with teriflunomide over 3 months.

	*N* = 100
Female, *n* (%)	64 (64.0)
Age at onset, median (range), years	26.9 (9.4, 56.8)
Age at teriflunomide initiation, median (range), years	32.0 (14.3, 57.0)
Disease duration before teriflunomide treatment, median (range), months	26.0 (0.5, 375.8)
Duration on teriflunomide treatment, median (range), months	19.7 (3.1, 51.7)
Number of relapses in 2 years before teriflunomide initiation, median (range)	1 (0, 3)
≥3, *n* (%)	15 (15.0)
<3, *n* (%)	85 (85.0)
Number of relapses in 1 year before teriflunomide initiation, median (range)	1 (0, 3)
≥2, *n* (%)	26 (26.0)
<2, *n* (%)	74 (74.0)
Number of relapses in initial 2 years of disease, median (range)	1 (0, 3)
≥2, *n* (%)	18 (18.0)
<2, *n* (%)	82 (82.0)
Regions involved at disease onset, *n* (%)	
Cerebrum	13 (13.0)
Brainstem/cerebellum	33 (33.0)
Spinal cord	26 (26.0)
Optic nerve	11 (11.0)
Multiple regions	17 (17.0)
Peak EDSS score of the first attack, median (range)	2.0 (1.0, 7.5)
Recovery from the first attack, *n* (%)	
Complete recovery	84 (84.0)
Incomplete recovery	16 (16.0)
Unmatched CSF OB, *n* (%)	63/81 (77.8)
Medications before teriflunomide, *n* (%) ^a^	
Leflunomide	2 (2.0)
Interferon-β	9 (9.0)
Mycophenolate mofetil	1(1.0)
Azathioprine	1(1.0)
EDSS score at teriflunomide initiation, mean ± SD, median (range)	1.3 ± 1.4, 1.0 (0.0, 4.0)
≥3, *n* (%)	10 (10.0)
<3, *n* (%)	90 (90.0)

a All patients had discontinued previous treatments over 6 months before teriflunomide initiation.

RRMS, relapsing–remitting multiple sclerosis; EDSS, Expanded Disability Status Scale; CSF, cerebrospinal fluid; OB, oligoclonal band; SD, standard deviation.

### Baseline MRI data

Among the 100 patients, 83 had baseline MRI performed at our center (**Figure 1**). A total of 71 (85.5%) patients showed ≥9 supratentorial T2 lesions, while 12 (14.5%) patients had 0–8 supratentorial lesions. A total of 45 (54.2%) patients had subtentorial T2 lesions with a median lesion number of 1 (0–9), and 20 (24.1%) patients had ≥3 subtentorial T2 lesions. The total T2 lesion volume per patient was 16.2 (0.1–85.2) mL, while the total brain volume was 1,416.0 (1,162.0–1,850.0) mL (**Table 2**, **Supplementary Table S1**).

**Table 2:** Baseline MRI features.

MRI measurements ^a^	
Number of supratentorial T2 lesions	
0–8, *n*/total (%)	12/83 (14.5)
≥9, *n*/total (%)	71/83 (85.5)
Number of subtentorial T2 lesions, median (range)	1 (0, 9)
0–2, *n*/total (%)	63/83 (75.9)
≥3, *n*/total (%)	20/83 (24.1)
Total T2 lesion volume, median (range), mL	16.2 (0.1, 85.2)
<30 mL, *n*/total (%)	59/83 (71.1)
≥30 mL, *n*/total (%)	24/83 (28.9)
Total brain volume, median (range), mL	1,416.0 (1,162.0, 1,850.0)
<1,500 mL, *n*/total (%)	59/83 (71.1)
≥1,500 mL, *n*/total (%)	24/83 (28.9)
Number of PRL, median (range)	3 (0, 30)
0, *n*/total (%)	14/76 (18.4)
1–3, *n*/total (%)	30/76 (39.5)
≥4, *n*/total (%)	32/76 (42.1)
Total PRL volume, median (range), mL	0.3 (0.0, 11.5)
<1 mL, *n*/total (%)	55/76 (72.4)
≥1 mL, *n*/total (%)	21/76 (27.6)
PRL/T2 lesion volume, median (range), %	2.3 (0.0, 49.0)
<1.0%, *n*/total (%)	25/76 (32.9)
1.0%–4.0%, *n*/total (%)	24/76 (31.6)
≥4.0%, *n*/total (%)	27/76 (35.5)

a A total of 83 patients had baseline MRI performed at our center, in which T2 lesion number, T2 lesion volume, and total brain volume were calculated. Among the 83 patients, 76 had baseline SWI, with PRL number, PRL volume, and PRL/T2 lesion volume percentage calculated.

MRI, magnetic resonance imaging; SWI, susceptibility-weighted imaging; PRL, paramagnetic rim lesion.

In the 76 patients who had baseline SWI (**Figure 1**), the median number of PRL per patient was 3 (0–30). A total of 62 (81.6%) patients had at least 1 PRL, and 32 (42.1%) patients had ≥ 4 PRLs. The total volume of PRL per patient was 0.3 (0.0–11.5) mL, accounting for 2.3% (0%–49.0%) of the total T2 lesion burden (**Table 2**, **Supplementary Table S2**).

There was no significant difference in baseline clinical features between patients with PRL ≥ 4 and those with PRL< 4 (**Supplementary Table S3**), while baseline EDSS score was slightly higher in patients with PRL volume ≥ 1 mL than in those with PRL volume <1 mL (1.3 vs. 1.0, *p* = 0.025) (**Supplementary Table S4**).

### Effectiveness

#### ARR

**Figure 2:**
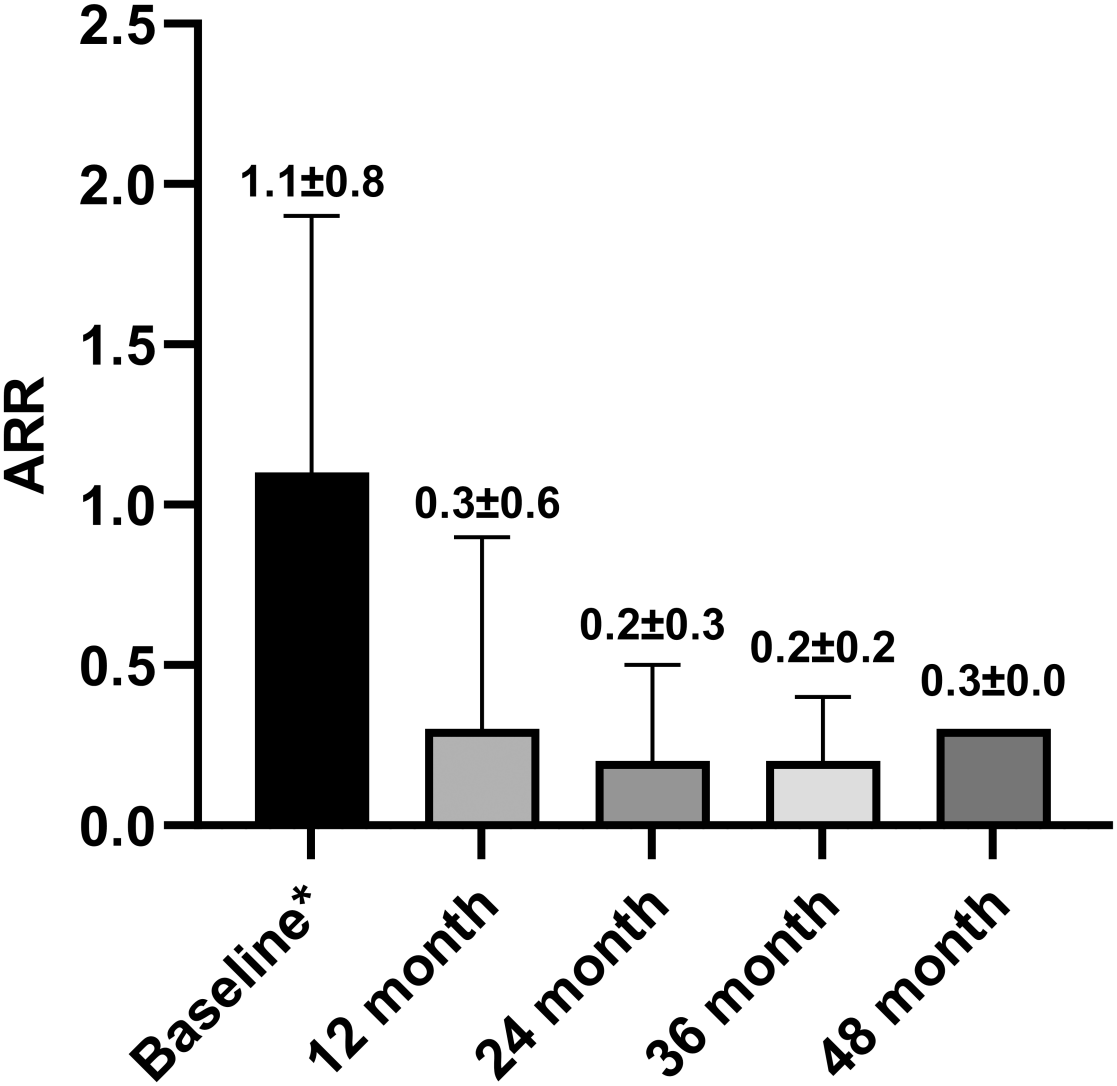
ARR before teriflunomide initiation and in 12, 24, 36, and 48 months after teriflunomide initiation. Means and SDs were displayed. ARR, Annualized relapse rate; SD, standard deviation. * One year before teriflunomide initiation.

A total of 96 patients treated over 6 months were included in the analysis (**Figure 1**). The ARR decreased from 1.1 ± 0.8 in the 12 months before teriflunomide initiation to 0.3 ± 0.5 after treatment (*p* < 0.001), with a 72.7% reduction (**Table 3**, **Figure 2**).

**Table 3:** Effectiveness of teriflunomide in the 96 patients treated over 6 months.

Effectiveness measures		
ARR before treatment ^a^, mean ± SD	1.1 ± 0.8	***p* < 0.001**
ARR after treatment, mean ± SD	0.3 ± 0.5	
EDSS at teriflunomide initiation, median (range)	1.0 (0.0, 4.0)	*p* = 0.087
EDSS at last follow-up after treatment, median (range)	1.0 (0.0, 4.0)	
No clinical relapse (*n* = 96)		
12 months, *n* (%)	66 (80.8)^b^	
24 months, *n* (%)	36 (65.1)	
No CDW (*n* = 96)		
12 months, *n* (%)	73 (91.0)	
24 months, *n* (%)	44 (80.2)	
No PIRA (*n* = 96)		
12 months, *n* (%)	73 (95.4)	
24 months, *n* (%)	43 (88.8)	
No RAW (*n* = 96)		
12 months, *n* (%)	73 (95.4)	
24 months, *n* (%)	43 (90.3)	
No MRI activity (*n* = 83^c^)		
12 months, *n* (%)	56 (75.2)	
24 months, *n* (%)	29 (57.7)	
NEDA3 achieved (*n* = 91^d^)		
12 months, *n* (%)	55 (64.1)	
24 months, *n* (%)	28 (43.8)	

a One year before teriflunomide initiation.

b Cumulative survival rate was calculated by multiplying the survival rate at each time point, taking into account the effect of censored data.

c A total of 83 patients were with baseline MRI performed at our center.

d A total of 13 patients were without baseline MRI, but 8 of them experienced clinical relapses or EDSS worsening, which were sufficient to determine NEDA3 failure; thus, 91 patients were included in the analysis of NEDA3.

ARR, annualized relapse rate; SD, standard deviation; EDSS, Expanded Disability Status Scale; CDW, confirmed disability worsening; PIRA, progression independent of relapse activity; RAW, relapse-associated worsening; MRI, magnetic resonance imaging; NEDA, no evidence of disease activity.

The p-values lower than 0.05 are in bold type.

**Figure 3:**
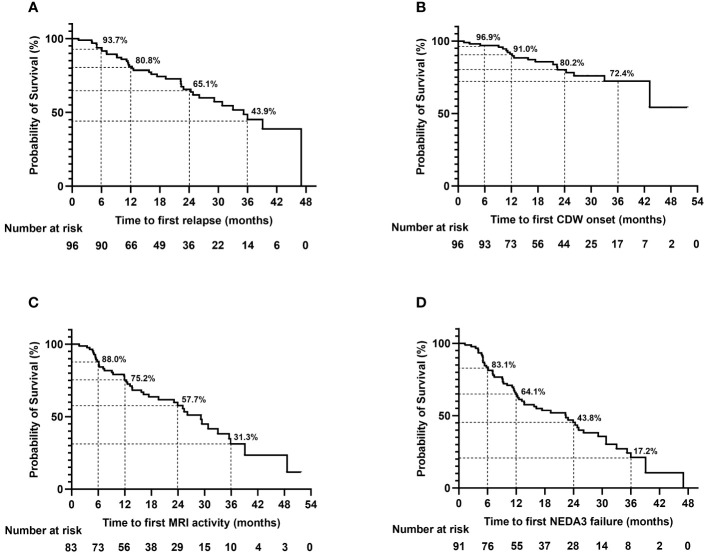
Time to first relapse **(A)**, CDW onset **(B)**, MRI activity **(C)**, and NEDA3 failure **(D)** during teriflunomide treatment. The time to event analyses were performed with the Kaplan–Meier method. MRI activity refers to appearance of new/enlarging T2 lesion or T1 gadolinium-enhancing lesion. CDW, confirmed disability worsening; MRI, magnetic resonance imaging; NEDA, no evidence of disease activity.

Forty-five relapses of 37 patients were recorded during teriflunomide treatment, the remaining patients (61.5%) were free of relapse till the last follow-up. The median time to first relapse was 12.6 (1.3–47.0) months (**Table 3**, **Figure 3A**).

#### EDSS worsening

**Figure 4:**
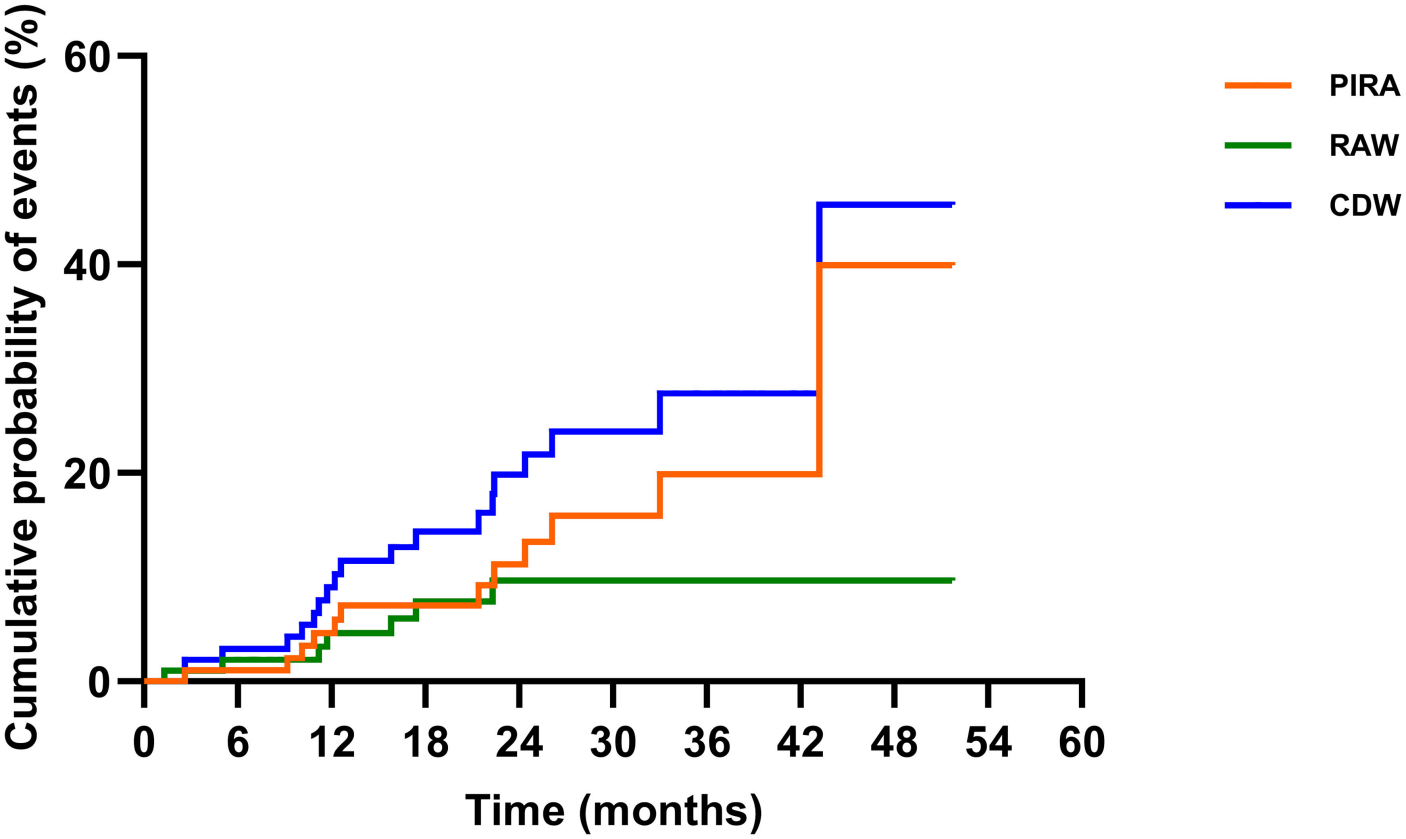
The contribution of PIRA in CDW. Disability was characterized using EDSS scores. CDW was based on EDSS and defined by an increase in EDSS (≥1.0 point for patients with a baseline EDSS of 0–5.0 and by 0.5 points for patients with a baseline EDSS of ≥5.5) confirmed by an EDSS assessment 6 months apart from the onset of the worsening, and sustained till the last available visits. RAW was a CDW event with an EDSS worsening onset within 90 days from the onset of a relapse. PIRA was defined as a CDW event with either no prior relapse or a EDSS worsening onset more than 90 days after the start date of the last relapse, and no relapse occurs within 30 days before or after the EDSS worsening confirmation at 6 months. The Kaplan–Meier method was applied to present the relative contributions of composite PIRA and RAW to CDW. EDSS, Expanded Disability Status Scale; PIRA, progression independent of relapse activity; RAW, relapse-associated worsening; CDW, confirmed disability worsening.

The EDSS scores were comparable before treatment and at the last follow-up [1.0 (0.0–4.0) vs. 1.0 (0.0–4.0), *p* = 0.087)]; 80.2% (77/96) of the patients were free of CDW throughout the observation. Nineteen patients developed CDW: 12 (63.2%) patients experienced PIRA, while 7 (36.8%) patients had relapse-associated worsening (RAW) (**Figure 4**). The median time to first CDW was 18.6 (1.3–43.2) months, and time to first PIRA was 21.4 (2.6–43.2) months (**Table 3**, **Figure 3B**).

During treatment, 9 (9.4%) patients converted to secondary progressive MS (SPMS) according to the definition raised by Lorscheider et al. (28), and the median time to SPMS was 22.4 (9.2–43.2) months. All of them discontinued teriflunomide and switched to other DMTs (six siponimod, two fingolimod, and one rituximab). After exchanging DMTs, all of them remained stable without further EDSS worsening.

#### MRI activity

Among the 83 patients with baseline MRI, 42 (50.6%) patients had at least once new/enlarging T2 or T1 Gd-enhancing lesions on serial MRI during teriflunomide treatment. The median time to first MRI activity was 12.5 (1.7–48.6) months (**Table 3**, **Figure 3C**).

#### NEDA3

NEDA3 was evaluated in the 83 patients with baseline MRI, and another 8 patients, despite without having baseline MRI, experienced clinical relapses and EDSS worsening, which were sufficient to judge NEDA3 (**Figure 1**).

Overall, 37.4% (34/91) achieved NEDA3 throughout the observation. Time to first NEDA3 failure was 11.5 (1.3–39.1) months. However, the proportion of NEDA3 failure increased with the extension of follow-up. At 12, 24, and 36 months of treatment, 64.1%, 43.8%, and 17.2% of the patients fulfilled NEDA3 (**Table 3**, **Figure 3D**). The most important contributor to NEDA3 failure was MRI activity.

### Factors associated with clinical relapse

A total of 76 patients with complete baseline MRI data (including SWI) were included to identify baseline factors associated with patient outcome during teriflunomide treatment (**Figure 1**).

For clinical relapse, univariate analysis identified that baseline PRLs ≥ 4 (OR = 3.73, 95% CI = 1.38–10.10, *p* = 0.009), subtentorial T2 lesions ≥ 3 (OR = 4.50, 95% CI = 1.53–13.25, *p* = 0.006), and total T2 lesion volume ≥ 30 mL (OR = 5.25, 95% CI = 1.79–15.43, *p* = 0.003) were associated with an increased risk of relapse during teriflunomide treatment. However, none was significant in multivariate analysis (**Table 4**).

**Table 4:** Factors associated with clinical relapse during teriflunomide treatment (*n* = 76^a^).

Factors	Univariate analysis			Multivariate analysis	
	OR (95% CI)	*p*-value	Corrected *p*-value	OR (95% CI)	*p*-value
Age at onset	0.96 (0.90, 1.01)	0.120	0.236		
Male	2.09 (0.77, 5.69)	0.150	0.236		
Disease duration before treatment	1.00 (0.99, 1.01)	0.859	0.859		
Frequent relapses before treatment^b^	2.33 (0.76, 7.19)	0.140	0.236		
Subtentorial/spinal cord involved at onset	1.98 (0.63, 6.19)	0.242	0.296		
Incomplete recovery from the first attack	0.46 (0.12, 1.83)	0.272	0.299		
Baseline EDSS score ≥ 3	4.36 (0.74, 25.64)	0.103	0.236		
Baseline PRL number ≥ 4	3.73 (1.38, 10.10)	**0.009**	0.033	2.11 (0.66, 6.73)	0.209
Baseline subtentorial T2 lesion number ≥ 3	4.50 (1.53, 13.25)	**0.006**	0.033	2.50 (0.72, 8.61)	0.148
Baseline T2 lesion volume ≥ 30 mL	5.25 (1.79, 15.43)	**0.003**	0.033	2.40 (0.64, 9.06)	0.196
Baseline total brain volume < 1,500 mL	0.53 (0.19, 1.52)	0.239	0.296		

a Patients treated with teriflunomide ≥ 6 months and with baseline SWI data.

b Patients were considered having frequent relapses before treatment if they experienced at least two attacks 1 year before treatment initiation or at least three attacks 2 years before treatment initiation.

OR, odds ratio; CI, confidence interval; EDSS, Expanded Disability Status Scale; PRL, paramagnetic rim lesion.

The p-values lower than 0.05 are in bold type.

When total PRL volume was applied instead of PRL number (**Supplementary Table S5**), we found that PRL volume ≥ 1 mL was independently associated with clinical relapse (OR = 3.43, 95% CI = 1.07–10.96, *p* = 0.038).

### Factors associated with CDW

Multivariate analyses identified that longer disease duration before treatment (OR = 1.02, 95% CI = 1.00–1.04, *p* = 0.012) was associated with an increased risk of CDW during teriflunomide treatment, while higher number of PRL showed a risky trend (OR = 7.62, 95% CI = 0.69–85.63, *p* = 0.098) (**Table 5**).

**Table 5:** Factors associated with CDW during teriflunomide treatment (*n* = 76^a^).

Factors	Univariate analysis			Multivariate analysis	
	OR (95%CI)	*p*-value	Corrected *p*-value	OR (95%CI)	*p*-value
Age at onset	0.98 (0.91, 1.06)	0.676	0.676		
Male	5.44 (1.23, 24.10)	**0.026**	0.099	5.59 (0.55, 56.62)	0.145
Disease duration before treatment	1.01 (1.00, 1.02)	**0.039**	0.099	1.02 (1.00, 1.04)	**0.012**
Frequent relapses during the initial 2 years of disease^b^	3.22 (0.68, 15.24)	0.140	0.220		
Subtentorial/spinal cord involved at onset	0.43 (0.10, 1.77)	0.239	0.292		
Incomplete recovery from first attack	0.52 (0.06, 4.53)	0.553	0.608		
Baseline EDSS score ≥ 3	4.50 (0.70, 29.15)	0.115	0.211		
Baseline PRL number ≥ 4	6.27 (1.21, 32.62)	**0.029**	0.099	7.62 (0.69, 85.63)	0.098
Baseline subtentorial T2 lesion number ≥ 3	2.55 (0.61, 10.65)	0.199	0.274		
Baseline T2 lesion volume ≥ 30 mL	6.93 (1.55, 31.08)	**0.011**	0.099	2.48 (0.35, 17.74)	0.365
Baseline total brain volume < 1,500 mL	0.23 (0.06, 0.97)	**0.045**	0.099	0.83 (0.08, 8.14)	0.871

a Patients with teriflunomide treatment ≥ 6 months and with baseline SWI data.

b ≥2 relapses in the initial 2 years of MS disease.

OR, odds ratio; CI, confidence interval; CDW, confirmed disability worsening; EDSS, Expanded Disability Status Scale; PRL, paramagnetic rim lesion.

The p-values lower than 0.05 are in bold type.

When total PRL volume was applied, we observed that longer disease duration before treatment (OR = 1.02, 95% CI = 1.00–1.04, *p* = 0.014) and PRL volume ≥ 1 mL (OR = 13.68, 95% CI = 1.26–148.99, *p* = 0.032) were significantly associated with CDW (**Supplementary Table S6**).

### Factors associated with NEDA3 failure

Multivariate analysis indicated that younger onset age (OR = 0.94, 95% CI = 0.88–0.99, *p* = 0.039), frequent relapses during the initial 2 years of disease (OR = 13.40, 95% CI = 1.36–131.93, *p* = 0.026), and baseline PRL number ≥ 4 (OR = 4.24, 95% CI = 1.43–12.57, *p* = 0.009) were independent factors associated with NEDA3 failure during teriflunomide treatment (**Table 6**). PRL volume ≥ 1 mL (OR = 3.73, 95% CI = 0.99–14.12, *p* = 0.053) showed a similar association with NEDA3 failure (**Supplementary Table S7**).

**Table 6:** Factors associated with NEDA3 failure (*n* = 76^a^).

Factors	Univariate analysis			Multivariate analysis	
	OR (95% CI)	*p*-value	Corrected *p*-value	OR (95% CI)	*p*-value
Age at onset	0.94 (0.90, 0.99)	**0.035**	0.128	0.94 (0.88, 0.99)	**0.039**
Male	0.87 (0.33, 2.29)	0.773	0.945		
Disease duration before treatment	1.00 (0.99, 1.01)	0.449	0.674		
Frequent relapses during the initial 2 years of disease^b^	11.00 (1.34, 90.27)	**0.026**	0.128	13.40 (1.36, 131.93)	**0.026**
Subtentorial/spinal cord involved at onset	0.97 (0.35, 2.67)	0.951	0.962		
Incomplete recovery from first attack	1.03 (0.32, 3.32)	0.962	0.962		
Baseline EDSS score ≥ 3	4.21 (0.47, 37.92)	0.200	0.367		
Baseline PRL number ≥ 4	5.17 (1.93, 13.82)	**0.001**	0.011	4.24 (1.43, 12.57)	**0.009**
Baseline subtentorial T2 lesion number ≥ 3	3.00 (0.96, 9.38)	0.059	0.162	2.34 (0.64, 8.51)	0.199
Baseline T2 lesion volume ≥ 30 mL	2.41 (0.82, 7.13)	0.112	0.246		
Baseline total brain volume < 1,500 mL	1.44 (0.52, 4.00)	0.490	0.674		

a Patients with teriflunomide ≥ 6 months and with baseline SWI data.

b ≥2 relapses in the initial 2 years of MS disease.

OR, odds ratio; CI, confidence interval; NEDA, no evidence of disease activity; EDSS, Expanded Disability Status Scale; PRL, paramagnetic rim lesion; SWI, susceptibility-weighted imaging; MS, multiple sclerosis.

The p-values lower than 0.05 are in bold type.

### Change of PRL during treatment

**Figure 5:**
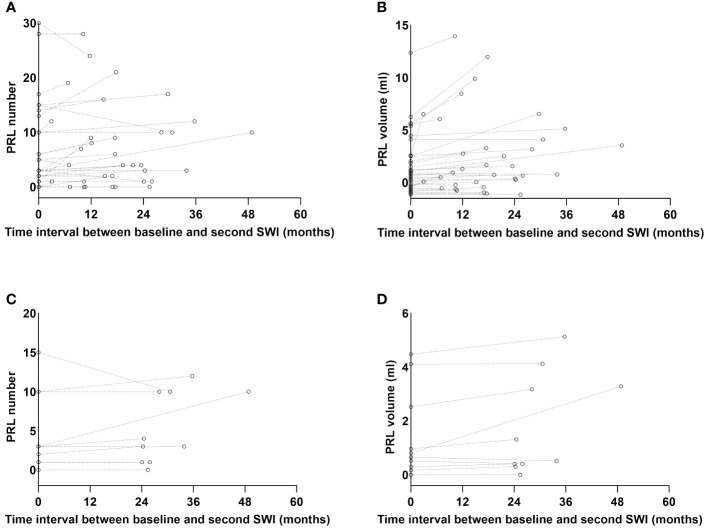
The PRL number and volume change over time. Besides a baseline SWI, 34 patients had a second SWI during teriflunomide treatment with a median interval of 17.2 (2.9–48.8) months. The median PRL number of the 34 patients increased from 3.0 (0.0–30.0) to 4.0 (0.0–28.0), *p* = 0.007 **(A)**, while the median PRL volume increased from 0.3 (0.0–12.4) to 0.7 (0.0–14.0) mL, *p* < 0.001 **(B)**. Among the 34 patients, 10 patients had a second SWI with an interval ≥24 months. In these 10 patients, PRL number was comparable between two SWI scans [3.0 (0.0–15.0) vs. 3.5 (0.0–12.0), *p* = 0.343] **(C)**, while the total PRL volume numerically increased from 0.4 (0.0–4.5) to 0.6 (0.0–5.1) mL, *p* = 0.051 **(D)**. PRL, paramagnetic rim lesion; SWI, susceptibility-weighted imaging.

Besides a baseline SWI, 34 patients had a second SWI during teriflunomide treatment. The time interval between the baseline and the second SWI was 17.2 (2.9–48.8) months. We therefore examined the change of PRL under teriflunomide treatment: the PRL number increased from 3.0 (0.0–30.0) at baseline to 4.0 (0.0–28.0) at second assessment (*p* = 0.007, **Figure 5A**), while the total PRL volume increased from 0.3 (0.0–12.4) to 0.7 (0.0–14.0) mL (*p* < 0.001, **Figure 5B**).

Since the half-life of PRLs was very long (29), we specifically examined their alterations in 10 patients who had two SWI scans over 24 (24.1–48.8) months apart. We discovered that the PRL number was comparable [3.0 (0.0–15.0) vs. 3.5 (0.0–12.0), *p* = 0.343, **Figure 5C**], while the total PRL volume numerically increased from 0.4 (0.0–4.5) to 0.6 (0.0–5.1) mL, *p* = 0.051 (**Figure 5D**).

### Safety and persistence

The safety profile of teriflunomide in the current study was generally comparable to previous reports (9–11) (**Table 7**). Overall, 69 patients (69%, 69/100) had AEs during treatment. The most common was hair thinning (63%), especially in the first 6 months after teriflunomide initiation. Leukopenia (16%), increase of liver enzymes (16%), urinary tract infection (11%), and weight loss (10%) were also common. Five patients discontinued treatment due to AEs while no one was subjected to reduced teriflunomide dose or became hospitalized because of serious AEs during follow-up.

**Table 7:** Adverse events in the 100 patients treated with teriflunomide over 3 months.

Adverse events	*n*	%
Hair shining	63	63
Leukopenia	16	16
Increase of liver enzymes	16	16
Urinary tract infection	11	11
Weight loss	10	10
Skin rash	9	9
Arthralgia	6	6
Aphthous ulcer	6	6
Diarrhea	4	4
Pruritus	3	3
Palpitation	2	2
Nausea and vomiting	1	1
Headache	1	1
Muscle pain	1	1
Abnormal menstruation	1	1
Patients with adverse events	69	69

**Figure 6:**
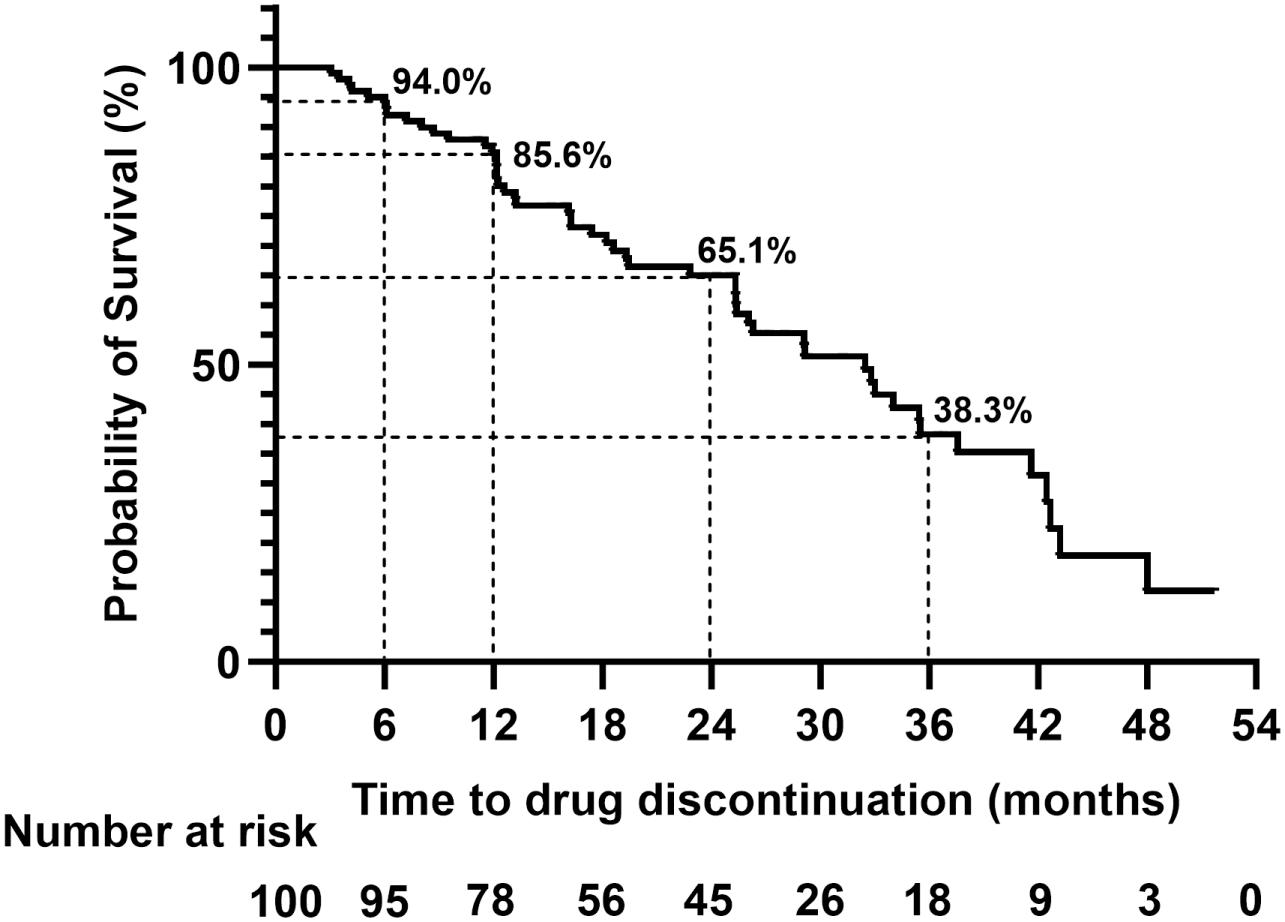
The persistence rate of the total cohort during teriflunomide treatment. At 6, 12, 24, and 36 months, the persistence rate of teriflunomide were 94%, 85.6%, 65.1%, and 38.3%, respectively.

At the last follow-up, 49 (49%) patients were still on teriflunomide after a median follow-up of 23.7 (6.0–51.7) months, whereas 51 (51%) patients had discontinued treatment due to various reasons with a median treatment time of 17.4 (3.1–48.0) months (**Table 8**). At month 24, the persistent rate of teriflunomide were 65.1% (**Figure 6**). A total of 47 patients switched to other DMTs after discontinuing teriflunomide (23 siponimod, 14 fingolimod, 7 rituximab, and 3 ofatumumab).

**Table 8:** Reasons for teriflunomide discontinuation.

Reasons for teriflunomide discontinuation ^a^	*n*	%
Clinical relapses	23	45.1
Disease progression	12	23.5
New/newly enlarged T2 or T1 Gd-enhanced lesions	6	11.8
Adverse events	5	9.8
Economic affordability	3	5.9
Pregnancy preparations	2	3.9

a Altogether, 51 patients discontinued treatment due to various reasons with a median treatment time of 17.4 (3.1–48.0) months.

Gd, gadolinium.

## Discussion

The main findings of the current study were as follows. First, teriflunomide markedly reduced ARR from 1.1 ± 0.8 before treatment to 0.3 ± 0.5 after treatment with a 72.7% reduction, while the EDSS scores remained stable. Second, the proportion of NEDA3 decreased with the extension of treatment time; at months 12, 24, and 36, 64.1%, 43.8%, and 17.2% of the patients achieved NEDA3, with MRI activity being the most common reason for NEDA3 failure. Third, higher baseline PRL volume (≥1 mL) or higher baseline PRL number (≥4) was significantly associated with the increased risks of EDSS worsening and NEDA3 failure in the context of teriflunomide treatment. Finally, the PRL number and volume seemed to not be alleviated by teriflunomide within the time range of our study.

In two randomized, double-blind, placebo-controlled phase 3 studies in patients with relapsing MS, teriflunomide 14 mg once daily demonstrated significant efficacy over 2 years in reducing ARR and risk of 12-week CDW (5, 6). The performance of teriflunomide was further confirmed by recent real-world studies from China (9–11). Compared to before treatment, ARR reductions were 78.2%–79.7% after treatment and EDSS scores were stabilized (9–11). The 12-month NEDA3% was reported to be 79% (9, 10). The current study yields basically similar effectiveness outcomes. However, the significant ARR decrease observed in the current study may partly be attributed to “regression to mean”—the natural variation or chance that is difficult to avoid in a small-sample real-world study without a control group, which counts for a limitation of our study.

Besides ARR, we reported time to event data (time to first relapse, first CDW, first MRI activity, and first NEDA3 failure) over an observation period up to 51.7 months. In addition to a 12-month NEDA3% of 64.1%, we reported 24- and 36-month NEDA3% to be 43.8% and 17.2%, with MRI activity being the main reason of NEDA3 failure. This obvious decreasing trend of NEDA3% strengthens the necessity of routine follow-up of patients on platform DMT like teriflunomide, even if they are stable for the initial year of therapy. However, defining treatment response is complex in the real world. From the perspective of ARR, teriflunomide yields good effectiveness as it keeps ARR in a continuous low level (**Figure 2**). To date, ARR is still the primary endpoint of most MS drug trials. Compared to ARR, NEDA3, a composite indicator of three dimensions, is more difficult to fulfill. One new MRI lesion (either clinical or subclinical) leads to NEDA3 failure. However, even if treated with DMT, the emergence of new lesions is virtually inevitable in most patients, especially in patients receiving platform DMT such as teriflunomide. The longer the follow-up time, the higher the probability of new lesions appearing. Therefore, it is necessary to carefully weigh the treatment strategies after NEDA3 failure in complicated clinical settings. At least, NEDA3 failure is not a mandatory condition for dressing treatment change.

In studies of teriflunomide from Western countries (30–37), patients included were often older, with longer disease duration at teriflunomide initiation, and with a minority of the patients being treatment naïve. In our study, patients were younger [32.0 (14.3–57.0) years], with a shorter disease duration [26.0 (0.5, 375.8) months] and mostly treatment naïve. Moreover, population pharmacokinetics of teriflunomide in Chinese patients might be different from that in Caucasian patients, probably due to ABCG2 polymorphisms (38). Therefore, our study and other Chinese studies stand for the real-life performance of teriflunomide in treatment-naïve Chinese patients with a shorter disease duration. The extrapolation of the current findings should be cautious.

The safety profile of teriflunomide in ours was comparable to other Chinese studies (9–11), but the incidence of AEs was higher in Chinese patients (69%–69.6%) than in Caucasian patients (35.7%–38%). Hair thinning, leukopenia, and increase in the number of liver enzymes were commonly reported in China, while gastrointestinal symptom (especially diarrhea) was more frequent in Western countries (9, 30, 34).

We specifically explored the relevance of PRL during teriflunomide treatment. PRLs have iron-laden pro-inflammatory microglia/macrophages at lesion edges inducing a rim of decreased signal in SWI, which are the site of ongoing inflammation, demyelination, and sustained axonal damage (12–17). PRL is considered a subset of chronic active MS lesion, and a major determinant of long-term disability progression (18–20). Studies have provided evidence that PRLs expand over years and can occur throughout the disease course, including in patients with preclinical disease (15, 17, 39, 40). Herein, we first reported the incidence, number, and volume of PRL in Chinese patients with RRMS. In the current cohort, 81.6% patients had at least 1 PRL, 39.5% had 1–3 PRLs, and 42.1% had ≥4 PRLs. The percentage of patients with PRL was higher than those reported (52%–66.7%) in previous investigations published from 2013 to 2022 (16, 19, 41–43), which may be partly explained by differences in patients’ ethnics, cohort clinical feature, MRI instrument or parameters, and not yet unified criteria for identifying PRL. Additionally, we reported the total volume of PRL per patient to be 0.3 (0.0–11.5) mL, accounting for 2.3% (0.0%–49.0%) of the total T2 lesion volume (**Table 2**).

To date, PRL has not been commonly used in routine clinical assessments and treatment decision-making. Previous studies showed that PIRA occurred more frequently in patients with ≥4 PRL on baseline brain MRI than in those with no PRL. A cross-sectional study of 192 MS patients revealed that patients younger than 50 years with ≥4 PRL had more severe motor disability and lower cognitive performance than those without PRL (18, 19). In the current study, we found that baseline PRL volume ≥1 mL or PRL number ≥4 was significantly associated with increased risks of EDSS worsening or NEDA3 failure in the context of teriflunomide treatment. Besides PRL-related variables, “longer disease duration” was also associated with an increased risk of CDW; while “younger age at onset” and “frequent relapses in the initial 2 years of MS” were associated with an increased risk of NEDA3 failure. Unlike previous studies (32–34), we did not find baseline EDSS score to be associated with teriflunomide treatment outcome, perhaps due to the overall low baseline EDSS score 1.0 (0.0, 4.0) of the current cohort. Overall, our results provided information that patients with less PRL numbers or lower PRL volumes, less frequent relapses, shorter disease duration, and relatively older age may benefit more from teriflunomide. On the other hand, our results support the selection of high-efficacy DMT in patients with higher baseline PRL burden.

The effect of DMTs on PRL is largely unknown. Even MS patients treated with highly effective anti-CD20 therapies have unchanged PRL burden (18). As expected, we did not observe significant impact of teriflunomide on PRL number and volume, indicating a huge challenge in the field of MS therapy—targeting the iron-laden microglia at the edge of chronic active lesions, where the chronic active inflammation and axonal injury is compartmentalized behind a virtually closed blood–brain barrier. Inclusion of PRL as an exploratory endpoint in further MS clinical trials with longer observation time may shed light on this important issue.

This study has limitations. As a single-center observational study, selection bias cannot be avoided. However, this facilitates fine MRI follow-ups with unified parameters. The number of included patients was small, different from Western countries; MS is rather rare in China. Future multi-center studies with a large sample size and a longer follow-up are thus warranted.

## Conclusion

Our study demonstrated that teriflunomide is effective in Chinese patients with RRMS. We also provided a profile of patients that may gain more benefits from teriflunomide, and revealed the potential value of PRL in treatment decision-making.

## Data availability statement

The raw data supporting the conclusions of this article will be made available by the authors, without undue reservation.

## Ethics statement

The studies involving humans were approved by the medical ethics committees of Huashan Hospital (HIRB-2020824). The studies were conducted in accordance with the local legislation and institutional requirements. The participants provided their written informed consent to participate in this study.

## Author contributions

HT: Data curation, Formal Analysis, Investigation, Methodology, Writing – original draft, Writing – review & editing. XL: Data curation, Formal Analysis, Investigation, Methodology, Writing – original draft, Writing – review & editing. YL: Data curation, Formal Analysis, Investigation, Methodology, Writing – original draft, Writing – review & editing. FH: Data curation, Formal Analysis, Writing – original draft, Writing – review & editing. JZ: Data curation, Formal Analysis, Writing – original draft, Writing – review & editing. LZ: Data curation, Formal Analysis, Writing – original draft, Writing – review & editing. LY: Writing – review & editing. CZ: Writing – review & editing. CL: Writing – review & editing. QD: Writing – review & editing. HL: Conceptualization, Data curation, Formal Analysis, Investigation, Project administration, Supervision, Writing – review & editing. CQ: Conceptualization, Data curation, Formal Analysis, Investigation, Project administration, Supervision, Writing – review & editing.

## Glossary

**Table:**

MS	multiple sclerosis
CNS	central nervous system
DMTs	disease-modifying therapies
RCTs	randomized control trials
ARR	annualized relapse rate
NEDA	no evidence of disease activity
MRI	magnetic resonance imaging
PRL	paramagnetic rim lesion
SWI	susceptibility-weighted imaging
PIRA	progression independent of relapse activity
RRMS	relapsing–remitting multiple sclerosis
EDSS	Expanded Disability Status Scale
CDW	confirmed disability worsening
RAW	relapse-associated worsening
OR	odds ratio
CI	confidence interval
SD	standard deviation
IQR	interquartile range
CSF	cerebrospinal fluid
OB	oligoclonal band
