# Supplementary material for: Real-world experience of teriflunomide in relapsing multiple sclerosis: paramagnetic rim lesions may play a role
Source: Front Immunol. 2024 Mar 13;15:1343531. doi: 10.3389/fimmu.2024.1343531 (PMC10979358; doi:10.3389/fimmu.2024.1343531)
Supplement: Supplementary file 1 [file Table_1.docx]

**Supplementary materials**

|  | n=83 |
| --- | --- |
| Female, n (%) | 56 (67.5) |
| Age at onset, median (range), years | 26.8 (9.4, 56.8) |
| Age at teriflunomide initiation, median (range), years | 32.2 (14.3, 57.0) |
| Disease duration before teriflunomide treatment, median (range), months | 24.2 (0.5, 375.8) |
| Duration on teriflunomide treatment, median (range), months | 21.2 (6.0, 51.7) |
| Number of relapses in 2 years before teriflunomide initiation, median (range) | 1 (0, 3) |
| ≥ 3, n (%) | 13 (15.7) |
| < 3, n (%) | 70 (84.3) |
| Number of relapses in 1 year before teriflunomide initiation, median (range) | 1 (0, 3) |
| ≥ 2, n (%) | 19 (22.9) |
| < 2, n (%) | 64 (77.1) |
| Number of relapses in the initial 2 years after disease onset, median (range) | 1 (0, 3) |
| ≥ 2, n (%) | 13 (15.7) |
| < 2, n (%) | 70 (84.3) |
| Regions involved at disease onset, n (%) |  |
| Cerebrum | 12 (14.5) |
| Brainstem/cerebellum | 27 (32.5) |
| Spinal cord | 19 (22.9) |
| Optic nerve | 9 (10.8) |
| Multiple regions | 16 (19.3) |
| Peak EDSS score of the first attack, median (range) | 2.0 (1.0, 7.5) |
| Recovery from the first attack, n (%) |  |
| Complete recovery | 69 (83.1) |
| Incomplete recovery | 14 (16.9) |
| Unmatched CSF OB, n (%) | 55/70 (78.6) |
| Medications before teriflunomide, n (%) ^a^ |  |
| Leflunomide | 2 (2.4) |
| Interferon-β | 8 (9.6) |
| Mycophenolate mofetil | 1(1.2) |
| EDSS score at teriflunomide initiation, mean±SD, median (range) | 1.1 ± 1.2, 1.0 (0.0, 4.0) |
| ≥ 3, n (%) | 6 (7.2) |
| < 3, n (%) | 77 (92.8) |

**Supplementary Table 1 Demographic and clinical data of the 83 patients with baseline MRI performed at our center**

^a^ All these patients had discontinued previous treatments over six months before teriflunomide initiation.

Abbreviations: MRI, magnetic resonance imaging; EDSS, Expanded Disability Status Scale; CSF, cerebrospinal fluid; OB, oligoclonal band; SD, standard deviation.

**Supplementary Table 2 Demographic and clinical data of the 76 patients with baseline SWI performed at our center**

|  | n=76 |
| --- | --- |
| Female, n (%) | 51 (67.1) |
| Age at onset, median (range), years | 26.4 (9.4, 56.8) |
| Age at teriflunomide initiation, median (range), years | 32.2 (14.3, 57.0) |
| Disease duration before teriflunomide treatment, median (range), months | 24.4 (0.5, 244.6) |
| Duration on teriflunomide treatment, median (range), months | 20.7 (6.0, 51.7) |
| Number of relapses in 2 years before teriflunomide initiation, median (range) | 1 (0, 3) |
| ≥ 3, n (%) | 11 (14.5) |
| < 3, n (%) | 65 (85.5) |
| Number of relapses in 1 year before teriflunomide initiation, median (range) | 1 (0, 3) |
| ≥ 2, n (%) | 16 (21.1) |
| < 2, n (%) | 60 (78.9) |
| Number of relapses in the initial 2 years after disease onset, median (range) | 0.5 (0, 3) |
| ≥ 2, n (%) | 12 (15.8) |
| < 2, n (%) | 64 (84.2) |
| Regions involved at disease onset, n (%) |  |
| Cerebrum | 11 (14.5) |
| Brainstem/cerebellum | 25 (32.9) |
| Spinal cord | 16 (21.1) |
| Optic nerve | 9 (11.8) |
| Multiple regions | 15 (19.7) |
| Peak EDSS score of the first attack, median (range) | 2.0 (1.0, 7.5) |
| Recovery from the first attack, n (%) |  |
| Complete recovery | 63 (82.9) |
| Incomplete recovery | 13 (17.1) |
| Unmatched CSF OB, n (%) | 51/65 (78.5) |
| Medications before teriflunomide, n (%) ^a^ |  |
| Leflunomide | 2 (2.6) |
| Interferon-β | 8 (10.5) |
| Mycophenolate mofetil | 1(1.3) |
| EDSS score at teriflunomide initiation, mean±SD, median (range) | 1.1 ± 1.2, 1.0 (0.0, 4.0) |
| ≥ 3, n (%) | 6 (7.9) |
| < 3, n (%) | 70 (92.1) |

^a^ All these patients had discontinued previous treatments over six months before teriflunomide initiation.

Abbreviations: SWI, susceptibility-weighted imaging; EDSS, Expanded Disability Status Scale; CSF, cerebrospinal fluid; OB, oligoclonal band; SD, standard deviation.

**Supplementary Table 3 Baseline demographic and clinical data of patients with PRL ≥ 4 and PRL < 4**

|  | PRL ≥ 4 (n=32) | PRL< 4 (n=44) | p value |
| --- | --- | --- | --- |
| Female, n (%) | 20 (62.5) | 31 (70.5) | 0.466 |
| Age at onset, median (range), years | 29.0 (13.6, 56.8) | 26.0 (9.4, 50.9) | 0.666 |
| Age at teriflunomide initiation, median (range), years | 32.2 (18.4, 57.0) | 32.1 (14.3, 51.6) | 0.821 |
| Disease duration before teriflunomide treatment, median (range), months | 25.9 (0.5, 168.4) | 18.2 (0.6, 244.6) | 0.336 |
| Duration on teriflunomide treatment, median (range), months | 24.1 (6.0, 51.7) | 19.7 (6.0, 51.7) | 0.418 |
| Number of relapses in 2 years before teriflunomide initiation, median (range) | 1 (0, 3) | 1 (0, 3) | 0.534 |
| ≥ 3, n (%) | 7 (21.9) | 4 (9.1) | 0.186 |
| < 3, n (%) | 25 (78.1) | 40 (90.9) |  |
| Number of relapses in 1 year before teriflunomide initiation, median (range) | 1 (0, 3) | 1 (0, 3) | 0.925 |
| ≥ 2, n (%) | 8 (25.0) | 8 (18.2) | 0.472 |
| < 2, n (%) | 24 (75.0) | 36 (81.8) |  |
| Number of relapses in the initial 2 years after disease onset, median (range) | 1 (0, 3) | 0 (0, 3) | 0.344 |
| ≥ 2, n (%) | 6 (18.7) | 6 (13.6) | 0.546 |
| < 2, n (%) | 26 (81.3) | 38 (86.4) |  |
| Regions involved at disease onset, n (%) |  |  |  |
| Cerebrum | 4 (12.5) | 7 (15.9) | 0.548 |
| Brainstem/cerebellum | 14 (43.8) | 11 (25.0) |  |
| Spinal cord | 5 (15.6) | 11 (25.0) |  |
| Optic nerve | 3 (9.4) | 6 (13.6) |  |
| Multiple regions | 6 (18.8) | 9 (20.5) |  |
| Peak EDSS score of the first attack, median (range) | 2.0 (1.0, 7.5) | 2.0 (1.0, 6.0) | 0.937 |
| Recovery from the first attack, n (%) |  |  |  |
| Complete recovery | 26 (81.3) | 37 (84.1) | 0.766 |
| Incomplete recovery | 6 (18.7) | 7 (15.9) |  |
| Unmatched CSF OB, n (%) | 19/25 (76.0) | 32/40 (80.0) | 0.705 |
| Medications before teriflunomide, n (%) ^a^ |  |  |  |
| Leflunomide | 1 (3.1) | 1 (2.3) |  |
| Interferon-β | 4 (12.5) | 4 (9.1) |  |
| Mycophenolate mofetil | 1(3.1) | 0 |  |
| EDSS score at teriflunomide initiation, median (range) | 1.0 (0.0, 4.0) | 1.0 (0.0, 3.5) | 0.116 |
| ≥ 3, n (%) | 5 (15.6) | 1 (2.3) | 0.077 |
| < 3, n (%) | 27 (84.4) | 43 (97.7) |  |

^a^ All these patients had discontinued previous treatments over six months before teriflunomide initiation.

Abbreviations: PRL, paramagnetic rim lesion; EDSS, expanded disability status scale; CSF, cerebrospinal fluid; OB, oligoclonal band.

**Supplementary Table 4 Baseline demographic and clinical data of patients with PRL volume**

|  | PRL ≥ 1ml (n=22) | PRL< 1ml  (n=54) | p value |
| --- | --- | --- | --- |
| Female, n (%) | 15 (68.2) | 36 (66.7) | 0.899 |
| Age at onset, median (range), years | 26.7 (13.6, 56.8) | 26.4 (9.4, 50.9) | 0.116 |
| Age at teriflunomide initiation, median (range), years | 32.6 (19.2, 57.0) | 32.0 (14.3, 51.6) | 0.766 |
| Disease duration before teriflunomide treatment, median (range), months | 29.4 (0.5, 168.4) | 19.8 (0.6, 244.6) | 0.300 |
| Duration on teriflunomide treatment, median (range), months | 24.1 (6.0, 43.2) | 19.7 (6.0, 51.7) | 1.000 |
| Number of relapses in 2 years before teriflunomide initiation, median (range) | 1 (0, 3) | 1 (0, 3) | 0.830 |
| ≥ 3, n (%) | 4 (18.2) | 7 (13.0) | 0.720 |
| < 3, n (%) | 18 (81.8) | 47 (87.0) |  |
| Number of relapses in 1 year before teriflunomide initiation, median (range) | 1 (0, 3) | 1 (0, 3) | 0.662 |
| ≥ 2, n (%) | 4 (18.2) | 12 (22.2) | 0.767 |
| < 2, n (%) | 18 (81.8) | 42 (77.8) |  |
| Number of relapses in the initial 2 years after disease onset, median (range) | 1 (0, 2) | 0 (0, 3) | 0.514 |
| ≥ 2, n (%) | 3 (13.6) | 9 (16.7) | 1.000 |
| < 2, n (%) | 19 (86.4) | 45 (83.3) |  |
| Regions involved at disease onset, n (%) |  |  |  |
| Cerebrum | 4 (18.2) | 7 (13.0) | 0.805 |
| Brainstem/cerebellum | 9 (40.9) | 16 (29.6) |  |
| Spinal cord | 4 (18.2) | 12 (22.2) |  |
| Optic nerve | 2 (9.1) | 7 (13.0) |  |
| Multiple regions | 3 (13.6) | 12 (22.2) |  |
| Peak EDSS score of the first attack, median (range) | 2.0 (1.0, 3.0) | 2.0 (1.0, 7.5) | 0.165 |
| Recovery from the first attack, n (%) |  |  |  |
| Complete recovery | 17 (77.3) | 46 (85.2) | 0.504 |
| Incomplete recovery | 5 (22.7) | 8 (14.8) |  |
| Unmatched CSF OB, n (%) | 14/19 (73.7) | 37/46 (80.4) | 0.529 |
| Medications before teriflunomide, n (%) ^a^ |  |  |  |
| Leflunomide | 1 (4.5) | 1 (1.9) |  |
| Interferon-β | 4 (18.2) | 4 (7.4) |  |
| Mycophenolate mofetil | 0 | 1 (1.9) |  |
| EDSS score at teriflunomide initiation, median (range) | 1.3 (0.0, 4.0) | 1.0 (0.0, 4.0) | **0.025** |
| ≥ 3, n (%) | 5 (22.7) | 1(1.9) | **0.007** |
| < 3, n (%) | 17 (77.3) | 53 (98.1) |  |

**≥ 1ml and < 1ml**

^a^ All these patients had discontinued previous treatments over six months before teriflunomide initiation.

Abbreviations: PRL, paramagnetic rim lesion; EDSS, expanded disability status scale; CSF, cerebrospinal fluid; OB, oligoclonal band.

**Supplementary Table 5 Factors associated with clinical relapse during teriflunomide treatment (n=76^a^)**

| Factors | Univariate analysis | | | Multivariate analysis |  |
| --- | --- | --- | --- | --- | --- |
|  | OR (95% CI) | p value | Corrected p value | OR (95% CI) | p value |
| Age at onset | 0.96 (0.90, 1.01) | 0.120 | 0.236 |  |  |
| Male | 2.09 (0.77, 5.69) | 0.150 | 0.236 |  |  |
| Disease duration before treatment | 1.00 (0.99, 1.01) | 0.859 | 0.859 |  |  |
| Frequent relapses before treatment^b^ | 2.33 (0.76, 7.19) | 0.140 | 0.236 |  |  |
| Subtentorial/spinal cord involved at onset | 1.98 (0.63, 6.19) | 0.242 | 0.296 |  |  |
| Incomplete recovery from the first attack | 0.46 (0.12, 1.83) | 0.272 | 0.299 |  |  |
| Baseline EDSS score ≥ 3 | 4.36 (0.74, 25.64) | 0.103 | 0.236 |  |  |
| Baseline PRL volume ≥ 1ml | 5.25 (1.79, 15.43) | **0.003** | 0.017 | 3.43 (1.07, 10.96) | **0.038** |
| Baseline subtentorial T2 lesion number ≥ 3 | 4.50 (1.53, 13.25) | **0.006** | 0.022 | 2.18 (0.61, 7.81) | 0.234 |
| Baseline T2 lesion volume ≥ 30ml | 5.25 (1.79, 15.43) | **0.003** | 0.017 | 2.70 (0.76, 9.58) | 0.124 |
| Baseline total brain volume < 1500ml | 0.53 (0.19, 1.52) | 0.239 | 0.296 |  |  |

^a^ Patients with teriflunomide treatment ≥ 6 months and with baseline SWI

^b^ Patients were considered having frequent relapses before treatment of they experienced at least 2 attacks one year before treatment initiation or at least 3 attacks two years before treatment initiation.

Abbreviations: OR, odds ratio; CI, confidence interval; EDSS, Expanded Disability Status Scale; PRL, paramagnetic rim lesion; SWI, susceptibility-weighted imaging.

**Supplementary Table 6 Factors associated with CDW during teriflunomide treatment(n=76^a^)**

| Factors | Univariate analysis | | | Multivariate analysis |  |
| --- | --- | --- | --- | --- | --- |
|  | OR (95%CI) | p value | Corrected p value | OR (95%CI) | p value |
| Age at onset | 0.98 (0.91, 1.06) | 0.676 | 0.676 |  |  |
| Male | 5.44 (1.23, 24.10) | **0.026** | 0.095 | 22.26 (0.92, 538.31) | 0.056 |
| Disease duration before treatment | 1.01 (1.00, 1.02) | **0.039** | 0.099 | 1.02 (1.00, 1.04) | **0.014** |
| Frequent relapses during the initial 2 years of disease^b^ | 3.22 (0.68, 15.24) | 0.140 | 0.22 |  |  |
| Subtentorial/spinal cord involved at onset | 0.43 (0.10, 1.77) | 0.239 | 0.292 |  |  |
| Incomplete recovery from first attack | 0.52 (0.06, 4.53) | 0.553 | 0.608 |  |  |
| Baseline EDSS score ≥ 3 | 4.50 (0.70, 29.15) | 0.115 | 0.211 |  |  |
| Baseline PRL volume ≥ 1ml | 6.93 (1.55, 31.08) | **0.011** | 0.061 | 13.68 (1.26, 148.99) | **0.032** |
| Baseline subtentorial T2 lesion number ≥ 3 | 2.55 (0.61, 10.65) | 0.199 | 0.274 |  |  |
| Baseline T2 lesion volume ≥ 30ml | 6.93 (1.55, 31.08) | **0.011** | 0.061 | 3.07 (0.44, 21.53) | 0.260 |
| Baseline total brain volume < 1500ml | 0.23 (0.06, 0.97) | **0.045** | 0.099 | 2.97 (0.17, 51.44) | 0.454 |

^a^ Patients with teriflunomide treatment ≥ 6 months and with baseline SWI

^b^ ≥ 2 relapses in the initial two years of MS disease.

Abbreviations: OR, odds ratio; CI, confidence interval; CDW, confirmed disability worsening; EDSS, Expanded Disability Status Scale; SWI, susceptibility-weighted imaging; PRL, paramagnetic rim lesion; MS, multiple sclerosis.

**Supplementary Table 7 Factors associated with NEDA3 failure during teriflunomide treatment(n=76^a^)**

| Factors | Univariate analysis | | | Multivariate analysis |  |
| --- | --- | --- | --- | --- | --- |
|  | OR (95%CI) | p value | Corrected p value | OR (95%CI) | p value |
| Age at onset | 0.94 (0.90, 0.99) | **0.035** | 0.139 | 0.93 (0.87, 0.99) | **0.021** |
| Male | 0.87 (0.33, 2.29) | 0.773 | 0.945 |  |  |
| Disease duration before treatment | 1.00 (0.99, 1.01) | 0.449 | 0.674 |  |  |
| Frequent relapses during the first 2 years of disease ^a^ | 11.00 (1.34, 90.27) | **0.026** | 0.139 | 16.34 (1.78, 149.83) | **0.013** |
| Subtentorial/spinal cord involved at onset | 0.97 (0.35, 2.67) | 0.951 | 0.962 |  |  |
| Incomplete recovery from first attack | 1.03 (0.32, 3.32) | 0.962 | 0.962 |  |  |
| Baseline EDSS score ≥ 3 | 4.21 (0.47, 37.92) | 0.200 | 0.367 |  |  |
| Baseline PRL volume ≥ 1ml | 3.32 (1.07, 10.32) | **0.038** | 0.139 | 3.73 (0.99, 14.12) | **0.053** |
| Baseline subtentorial T2 lesion number ≥ 3 | 3.00 (0.96, 9.38) | 0.059 | 0.162 | 2.06 (0.55, 7.73) | 0.286 |
| Baseline T2 lesion volume ≥ 30ml | 2.41 (0.82, 7.13) | 0.112 | 0.246 |  |  |
| Baseline total brain volume < 1500ml | 1.44 (0.52, 4.00) | 0.49 | 0.674 |  |  |

^a^ Patients with teriflunomide ≥ 6 months and with baseline SWI.

^b^≥ 2 relapses in the initial two years of MS disease.

Abbreviations: OR, odds ratio; CI, confidence interval; SWI, susceptibility-weighted imaging; NEDA, no evidence of disease activity; EDSS, Expanded Disability Status Scale; PRL, paramagnetic rim lesion; MS, multiple sclerosis.
